# Serum sclerostin in vascular calcification in CKD: a meta-analysis

**DOI:** 10.1080/0886022X.2023.2186151

**Published:** 2023-03-07

**Authors:** Yan Lin, Liman Mao, Siqi Chen, Canxin Zhou

**Affiliations:** Department of Nephrology, The Affiliated People’s Hospital, Ningbo University, Ningbo, China

**Keywords:** Cardiovascular events, chronic kidney disease, sclerostin, serum sclerostin, vascular calcification

## Abstract

Vascular calcification (VC) is recognized as a predictor of all-cause and CVD mortality in chronic kidney disease (CKD). VC in CKD is possibly associated with serum sclerostin. The study systematically investigated the role of serum sclerostin in VC in CKD. Following the Preferred Reporting Items for Systematic Review and Meta-Analysis Protocols, a systematic search was performed of the PubMed, Cochrane Library, and EMBASE databases from inception to 11 November 2022, to identify relevant eligible studies. The data were retrieved, analyzed, and summarized. The hazard ratios (HRs) and odds ratios (ORs) with their corresponding confidence intervals (CIs) were derived and pooled. Thirteen reports (3125 patients) met the inclusion criteria and were included. Sclerostin was associated with the presence of VC (pooled OR = 2.75, 95%CI = 1.81–4.19, *p* < 0.01) and all-cause mortality (pooled HR = 1.22, 95%CI = 1.19–1.25, *p* < 0.01) among patients with CKD, but with a decreased risk of cardiovascular events (HR = 0.98, 95%CI = 0.97–1.00, *p* = 0.02). This meta-analysis suggests that serum sclerostin is associated with VC and all-cause mortality among patients with CKD.

## GRAPHICAL ABSTRACT

**Figure:**
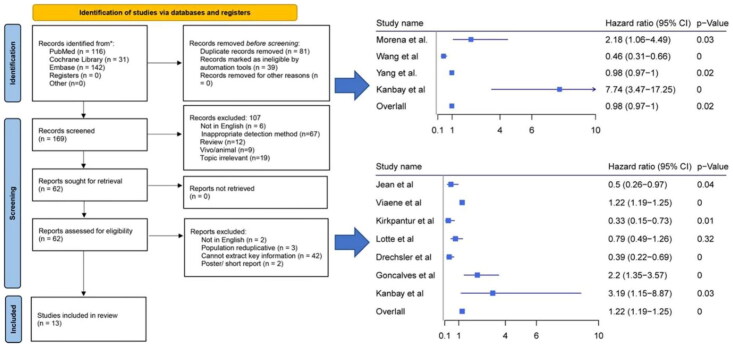

## Introduction

Chronic kidney disease (CKD) is characterized by the gradual loss of kidney function and is a major global health concern with high morbidity and mortality [1]. CKD is strongly associated with an increased risk of cardiovascular disease (CVD)-related mortality [2] and is an independent risk factor for CVD [3].

Vascular calcification (VC) is a predictor of all-cause and CVD-related mortality in patients with CKD [4]. VC in patients with CKD is associated with several traditional and nontraditional risk factors of CVD, including mineral metabolism disorders, for which serum sclerostin is a marker [5,6]. Indeed, sclerostin is a key osteocyte-derived soluble inhibitor of the Wnt signaling pathway and an indicator of bone formation that can suppress osteoblast differentiation and proliferation and promote osteoblast apoptosis [7,8]. Serum or circulating sclerostin is significantly increased among uremic patients and might promote CKD progression [9,10]. Nevertheless, the reported role of serum sclerostin in VC in CKD is inconsistent among reports [11–13]. Some investigations showed positive correlations, whereas others suggested no or negative correlations.

Therefore, this study systemically investigatedand summarized the association of serum sclerostin with VC and outcomes in patients with CKD.

## Materials and methods

The study was performed and reported according to the guidelines of the Preferred Reporting Items for Systematic Reviews and Meta-analyses (PRISMA) statement [14].

### Information sources and search strategy

A systematic search for the serum levels of sclerostin was performed using the PubMed, Cochrane Library, and EMBASE databases on 11 November 2022. The individual and joint key search terms included ‘serum sclerostin’, ‘vascular calcification’, and ‘chronic kidney disease’. No language or publication date filters were applied. A complete overview of the search strategy in the three databases is presented in the Supplementary Materials.

### Eligibility criteria

Articles were included if they met the following criteria:

1. Prospective and retrospective observational studies that evaluated the impact of serum sclerostin in vascular calcification in CKD;
2. Contained sufficient information detailing study design, patient characteristics, and outcomes;
3. Articles written only in English;
4. If the population was reported in duplicate, only the study providing more detailed information was included.

Articles meeting any of the following criteria were excluded:

1. Studies reporting based on cell lines or animals;
2. Reviews, case reports, abstracts, or posters for conferences, personal opinions, or book chapters;

### Data extraction and analysis

Two authors independently and systematically screened the titles and abstracts of the identified publications. The same two authors carried out the full-text assessment of the eligible reports independently. The data were extracted by the two authors independently using a customized and standardized form. They resolved any disagreements through discussions. If the two authors did not agree, a third author made the final decision. For each included study, the following data were extracted: the author and year of publication, country, sample size, sclerostin, follow-up time, anatomical structures, assays for measuring sclerostin, and the outcomes or associations.

### Risk of bias and quality assessment of the selected studies

Only observational studies were included in this meta-analysis. The assessment of the study quality was performed using the Newcastle-Ottawa Scale (NOS) [15]. Two authors (CGP and FA) independently evaluated the included studies. Disagreements were resolved by discussion to produce final scores. Using this tool, three domains were assessed: (1) selection of study groups (four points); (2) comparability of groups (two points); (3) ascertainment of exposure and outcomes (three points) for case-control and cohort studies, respectively.

### Statistical analysis

Hazard ratios (HRs) and odds ratios (ORs) with their corresponding confidence intervals (CIs) were derived from each included study and combined after log-transformation using a random effect model. Because the exposure values were treated as different forms (continuous and categorical), the effect sizes were pooled separately for studies that analyzed sclerostin levels as continuous values and those that categorized the sclerostin levels (e.g. below vs. above a cutoff value). Cochran’s Q statistics and the *I*^2^ statisticswere calculated to assess heterogeneity among studies. For higher values of the *I*^2^ index (an *I*^2^ index of 50% and 75% corresponds to moderate and high heterogeneity, respectively), sensitivity analysis and meta-regression were performed to explore the potential correlations between the study outcomes and patient numbers or study design [16]. In the sensitivity analysis, the cause of the high heterogeneity was investigated by the leave-one-out method, which involves sequentially excluding each study, one by one, to determine whether a single study was responsible for the high heterogeneity. The Begg rank correlation [17] and Egger weighted regression methods [18] were used to assess the publication bias (*p* < 0.05 was considered indicative of a statistically significant publication bias). Comprehensive Meta-analysis version 3.0 was used to generate the forest plots and statistical analyses. The Begg and Egger tests were performed using STATA 15.0 (Stata Corporation, College Station, TX). A two-sided *p*-value <0.05 was considered statistically significant.

## Results

### Search results

**Figure 1.**
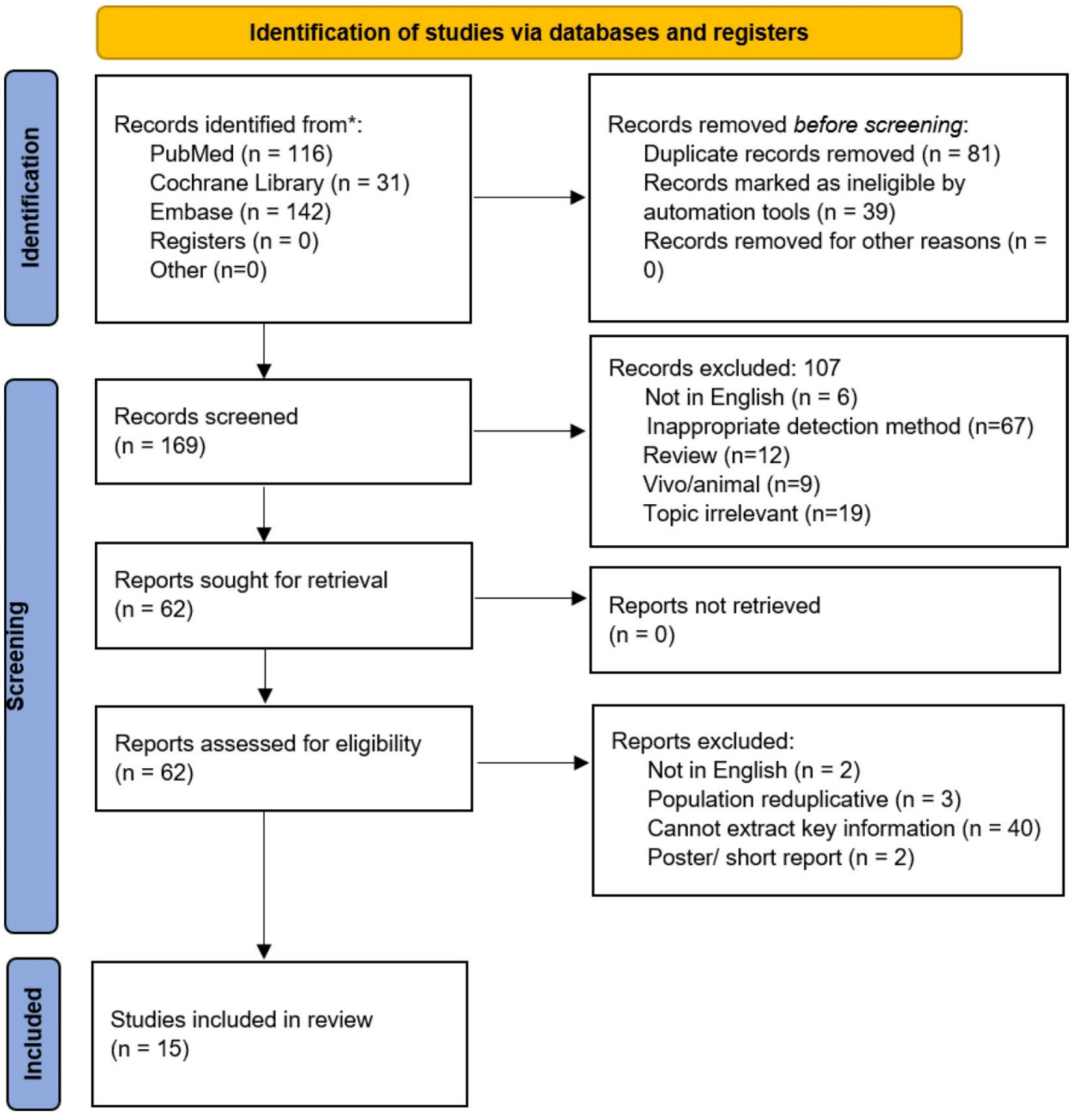
Flow chart of the study selection.

The initial screening of the electronic databases yielded 289 articles; 81 were duplicates, and 39 were marked as ineligible by automation tools; hence, 169 titles or abstracts were evaluated. After retrieving and reviewing 62 full-text reports, 15 studies fulfilled the inclusion criteria and were included in this systematic review and meta-analysis (Figure 1) [11,13,19–31].

### Characteristics of the included studies

An overview of the included studies and their characteristics is presented in Table 1. The 15 included studies were published between 2013 and 2021. A total of 3228 subjects were included. Most studies were based on either prospective or cross-sectional observational data. The studies were conducted in the USA, Netherlands, Turkey, Germany, Brazil, France, Sweden, China, Denmark, and Portugal. Sclerostin was measured using an enzyme-linked immunosorbent assay.

**Table 1.** Characteristics of the included studies.

Study	Country	Study design	Age (years)	Males (%)	Sclerostin	Enrolled patients	Follow-up time	Anatomical structures	Methods to detect VC
Claes et al. 2013 [11]	Belgium	Cross-sectional	59.7 ± 15	58	0.67 (0.3–0.8) ng/mL	241 ND-CKD	N/A	Coronary artery	Multidetector CT
Viaene et al. 2013 [19]	USA	Prospective	68 ± 13	60	110 (82–151) pmol/L	97 CKD3-4 patients	N/A	Abdominal aortic and common iliac artery, coronary artery, carotid artery	Multislice CT
Drechsler et al. 2014 [22]	Germany	Prospective	63 ± 14	44	1.24 ± 0.57 ng/mL	89 renal transplant recipients	N/A	Inferior epigastric artery	Biopsy and coronary artery CT
Gonçalves et al. 2014 [20]	Brazil	Prospective	42.3 ± 18.8	55	0.88 (0.54–1.55) ng/mL	161 CKD3-5 patients	N/A	Abdominal aortic artery	Abdominal lateral X-ray
Kanbay et al. 2014 [21]	Turkey	Prospective	47.0 (38.0 − 59.0)	86	63.5 (55.3–78.9) pmol/L	125 HD patients	2 years	Aorta	Chest radiography
Morena et al. 2015 [23]	France	Cross-sectional	69.0 (25.0–95.0)	59	0.92 (0.30–3.11) ng/mL	207 HD patients	N/A	Aorta	Lumbar radiography
Qureshi et al. 2015 [13]	Sweden	Cross-sectional	46 (24–62)	63	440 (230–857) pg/mL	268 prevalent RTRs	N/A	Coronary artery and aorta	Multislice CT
Yang et al. 2015 [24]	Taiwan	Prospective	56.9 ± 12.3/61.4 ± 9.9	49	73.1 ± 39.0 pmol/L	154 CKD1-5 patients	N/A	Aorta	Lumber X-ray
Evenepoel et al. 2015 [12]	Belgium	Prospective	53,0 (12,8)	61	0.84 (0.62–1.09) ng/mL	350 HD patients	4.4 years	Arteriovenous fistula	64-detector CT scanner
Jean et al. 2016 [25]	Belgium	Prospective	70.2 ± 14	43	1.9 ± 0.7 ng/mL	396 HD/HDF patients	Median: 2 years	N/A	N/A
Kirkpantur et al. 2016 [26]	Turkey	Prospective	52 ± 10/55 10 57 14	51	1519 ± 1378 pg/mL	673 PD/HD patients	N/A	Arteriovenous fistula	N/A
Wang et al. 2017 [28]	China	Prospective	58.3 ± 13.4	49	1007.62 ± 859.3 pg/mL	91 HD patients	>10 years	N/A	N/A
Lips et al. 2017 [27]	Netherlands	Prospective	64.1 (13.7)	62	139 (100–183) pmol/L	173 CKD3-5 (no dialysis) and 47 control patients	Median: 26 months	N/A	N/A
Jørgensen et al. 2018 [29]	Denmark	Prospective	54 (45–63)	68	N/A	157 late-stage CKD who were kidney transplantation candidates	Median: 3.7 years	Coronary arteries, thoracic aorta, and the aortic and mitral valves	Dual-source CT
Zhao et al. 2020 [30]	China	Cross-sectional	64 (51, 73)	51.4	46.76 pmol/L (IQR 30.18–67.56 pmol/L)	140 patients with stage 3–5 CKD	N/A	Carotid artery	B-mode Doppler ultrasound
Neto et al. 2021 [31]	Portugal	Cross-sectional	65.7 ± 9.8	78.6	2.32 ± 0.43 mg/dL	56 CKD patients (no dialysis)	N/A	Aortic wall calcification	Plain X-ray

Abbreviations: VC: vascular calcification; CKD: chronic kidney disease; HD: hemodialysis; CT: computed tomography; CVEs: cardiovascular events; ELISA: enzyme linked immunosorbent assay; NA: not available.

All studies used ELISA to measure sclerostin.

### Quality assessment of the studies

The NOS evaluation of the eligible studies is presented in Supplementary Table 1. All included studies had an acceptable quality. Six and nine studies were evaluated as 6 and 7 points, respectively.

### Relationship between sclerostin and VC

**Figure 2.**
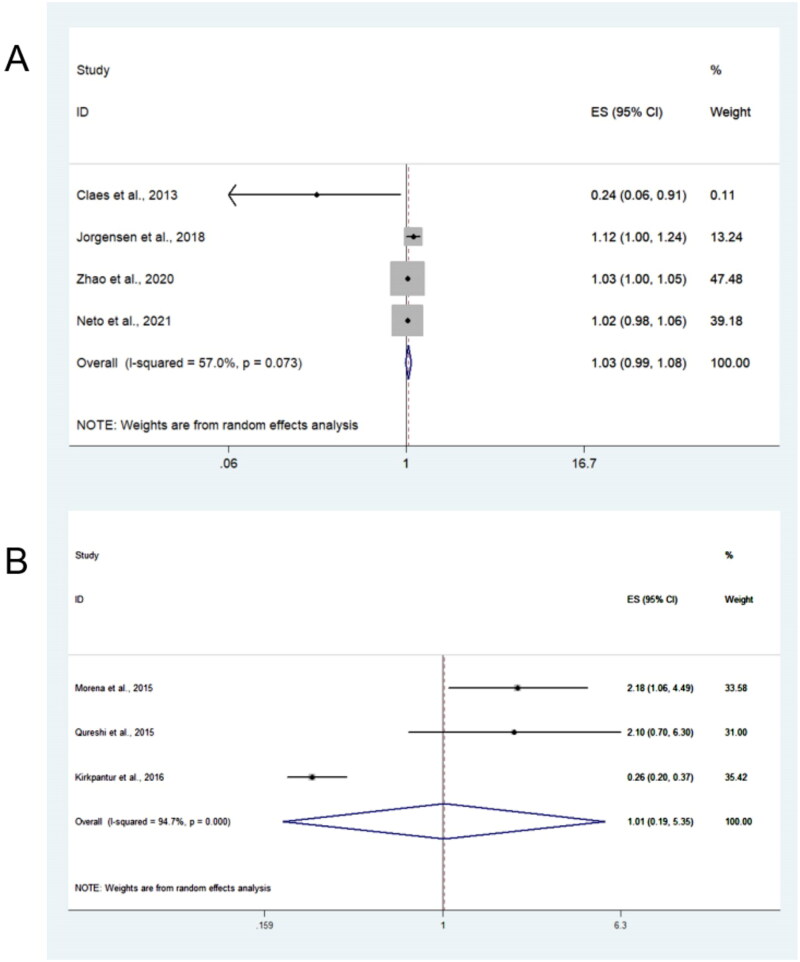
Forest plots for summarized relationship between sclerostin and VC. (a) pooled ORs for sclerostin ascontinuous values; (b) pooled ORs for sclerostin in categorical analyses.

Five studies reported the association of sclerostin with VC, and three examined the association using the continuous values of sclerostin (Figure 2(a)). Concerning the pooled relationship between sclerostin and VC, sclerostin was not significantly associated with VC in continuous values (pooled OR = 1.03, 95%CI = 0.99–1.08, *I*^2^ = 57.0%), and the results were consistent in the sensitivity analysis after omitting each study sequentially (Supplementary Figure S1). For categorical analyses of serum sclerostin, the association between sclerostin and VC was also non-significant (pooled OR = 1.01, 95%CI = 0.19–5.35, *I*^2^ = 94.7%, Figure 2(b)).In the sensitivity analysis, the association became significant if the study by Kirkpantur et al.in 2016 [26] was excluded (Supplementary Figure S2). Since the outcome of interest was arteriovenous fistula calcification in the Kirkpantur et al. study[26], it was excluded,and the pooled OR was 2.16 (95%CI = 1.18–3.94; Supplementary Figure S3).

**Figure 3.**
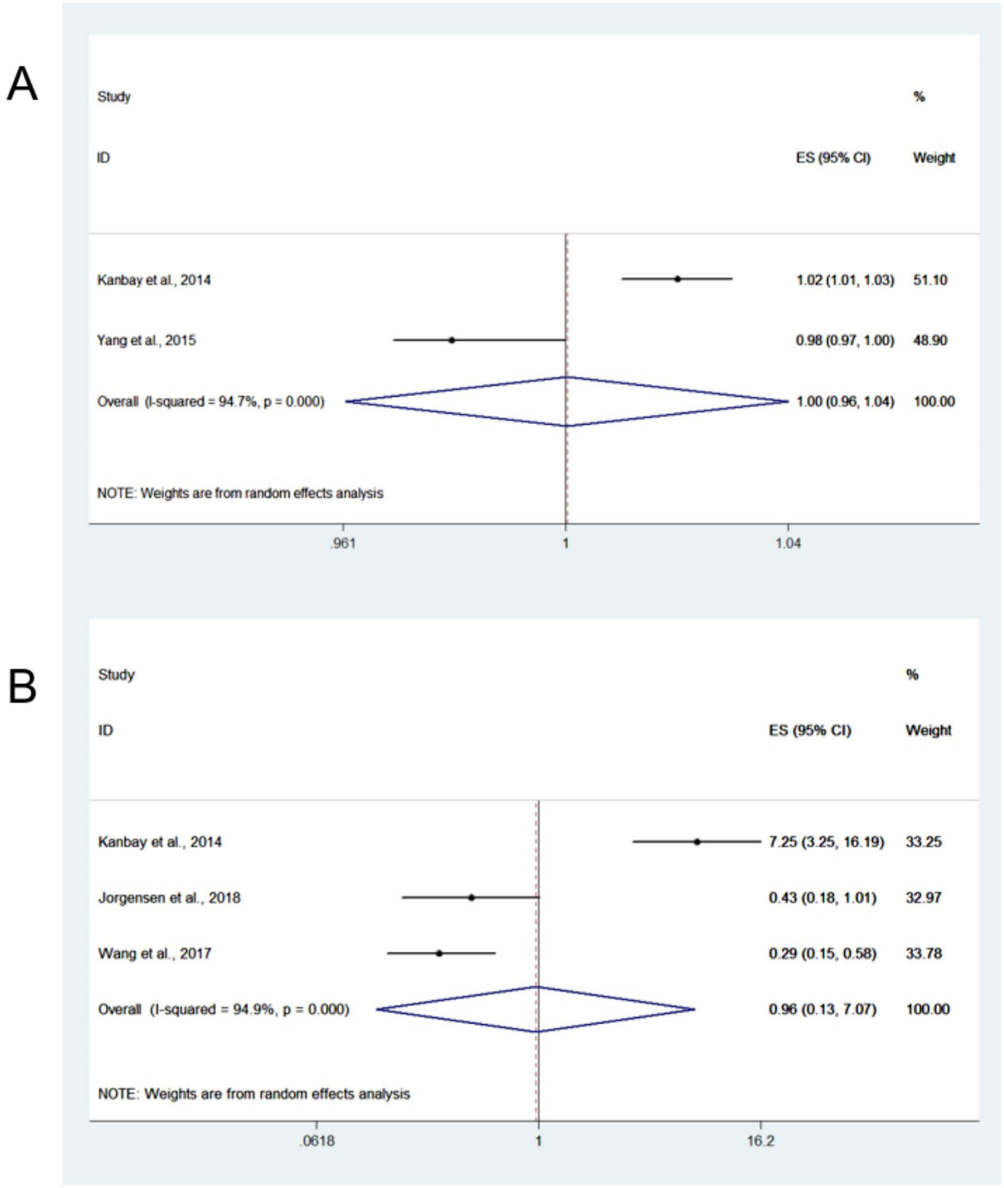
Forest plots for summarized relationship between sclerostin and cardiovascular events. (a) pooled HRs for sclerostin ascontinuous values; (b) pooled HRs for sclerostin in categorical analyses.

### Relationship between sclerostin and cardiovascular events

The relationship between sclerostin and cardiovascular events is shown in Figure 3. Five studies reported the association, and two reported HRs using continuous values. When summarizing the HRs, the overall HR was 1.00 (95%CI = 0.96–1.04) and 1.01 (95%CI = 0.19–5.35) for studies that used continuous (Figure 3(a)) and categorical (Figure 3(b)) analyses, respectively, with significant heterogeneity (*I*^2^ = 94.7%). We used the leave-one-out method to assess the robustness of our results in the sensitivity analysis (Supplementary Figure S4). When the study by Kanbay et al. [21] was excluded, a decreased heterogeneity was observed (*I*^2^ = 0%), and serum sclerostin was associated with the risk of cardiovascular events (pooled HR, 0.34; 95% CI, 0.20–0.57). The forest plot without Kanbay et al. [21] is presented in Supplementary Figure S5.

**Figure 4.**
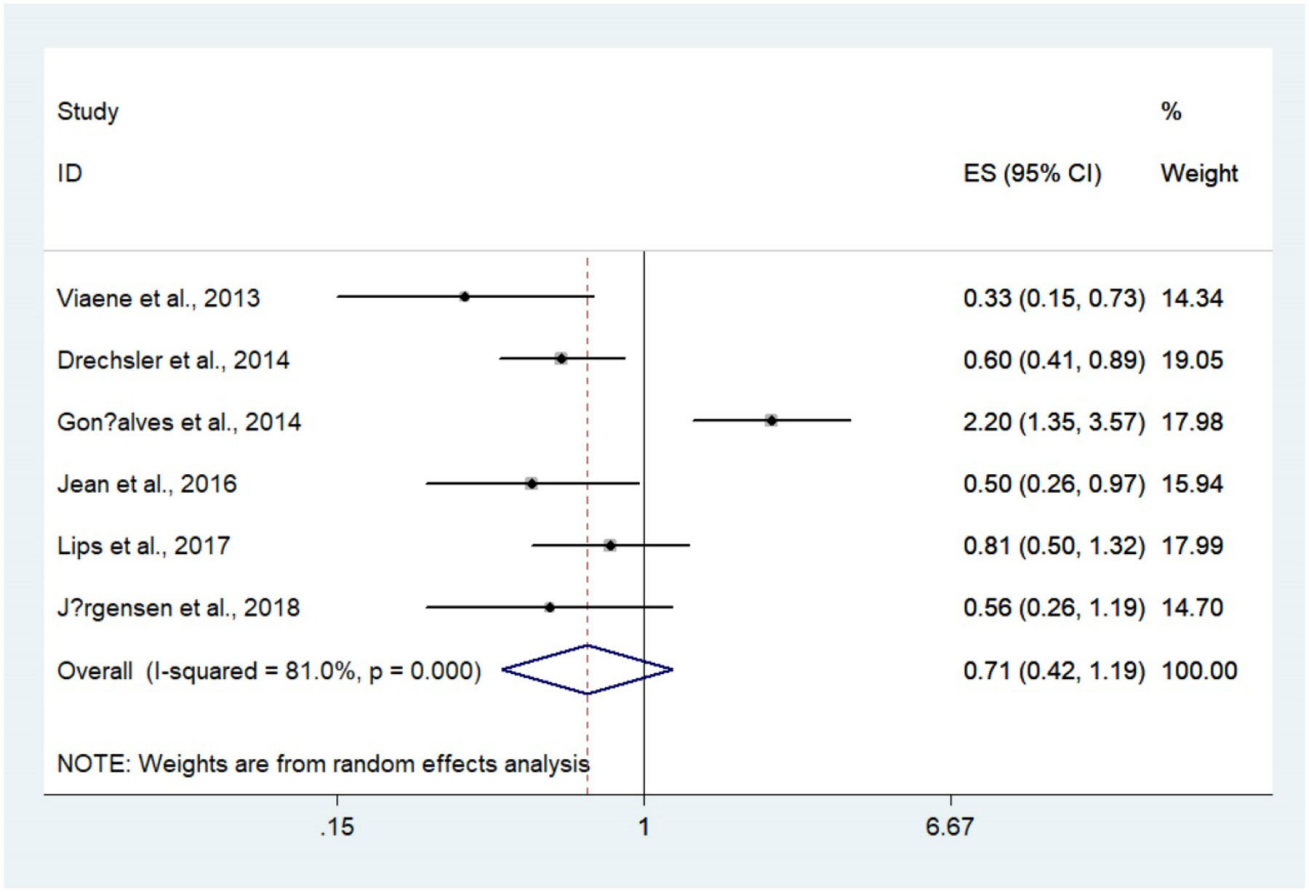
Forest plots for summarized relationship between sclerostin and all-cause mortality.

### Relationship between sclerostin and all-cause mortality

The relationship between sclerostin and all-cause mortality is presented in Figure 4. Six studies reported arelationship with HRs ranging from 0.33 to 2.20, and all these studies reported the associations using categorical analyses. When summarizing HRs, the pooled results showed that sclerostin was not associated with all-cause mortality (pooled HR = 0.71, 95%CI = 0.42–1.19, *p* < 0.01), with significant heterogeneity (*I*^2^ = 81.0%). When the study conducted by Gonçalves et al. [20] was excluded from the sensitivity analysis (Supplementary Figure S6), a decreased heterogeneity was seen (*I*^2^ = 0%). The forest plot omitting Gonçalves et al. [20] is presented in Supplementary Figure S7, and serum sclerostin was associated with all-cause mortality (pooled HR, 0.59; 95% CI, 0.46–0.76).

### Publication bias

The analysis did not observe potential publication bias among the included trials according to Begg rank correlation analysis and Egger weighted regression analysis (*p* > 0.050) (Supplementary Table S2).

## Discussion

To the authors’ knowledge, the present study is the first systematic review and meta-analysis study summarizing the association of serum sclerostin with VC and outcome in CKD patients. Thirteen studies (3125 subjects) were included and analyzed. The results suggest that serum sclerostin is associated with VC and all-cause mortality in patients with CKD. Increased serum sclerostin levels appear to be associated with decreased CVD events.

The classification of CKD was developed by the National Kidney Foundation Kidney Disease Outcomes Quality Initiative and is divided into five stages based on the levels of kidney function [32], which are determined by calculating the estimated glomerular filtration rate. VC was found to occur frequently in patients with CKD, and the incidence increases from CKD stages I to V [33], The mortality in the advanced stages of CKD remains unacceptably high, with large numbers of deaths caused by cardiovascular failure or dysfunction [34], The comorbidities of CKD include abnormal conditions of mineral bone metabolism and ectopic calcification, particularly VC, which is associated with an increased risk of CVD and all-cause mortality [35].

Sclerostin is a secreted glycoprotein possessing a C-terminal cysteine knot-like (CTCK) domain and sequence similarity to the differential screening-selected gene aberrative in neuroblastoma (DAN) family of bone morphogenetic protein (BMP) antagonists. Sclerostin is produced primarily by osteocytes but is also expressed in other tissues [36] and has anti-anabolic effects on bone formation [37]. The development of CVD involves many steps and factors, but one of the crucial events is the transdifferentiation of the vascular smooth muscle cells (VSMCs) [38], and transdifferentiated VSMCs can actively deposit hydroxyapatite in the medial layers of arteries, participating in the development of VC [39]. The Wnt/β-catenin and PPARγ pathways are involved in the fate of the transdifferentiated VSMCs [40,41]. Sclerostin is an inhibitor of the canonical Wnt/β-catenin pathway in the bone formation process and can induce adipocyte differentiation [42]. Some studies showed decreased mortality with high sclerostin levels in patients on hemodialysis [19,27]. On the other hand, although sclerostin expression was once thought to be confined to osteocytes, it is now known that sclerostin can be expressed by VSMCs adjacent to VC areas [43–45]. In addition, patients with CKD and VC have higher sclerostin levels than those without VC [11,43,44].Furthermore, sclerostin is eliminated by the kidney, and decreased kidney function will increase sclerostin levels [46]. Therefore, excess sclerostin produced in vessels with VC might prevent the progression of VC [47]. Excess sclerostin has also been suggested to be involved in the progression of CKD and related bone mineral disorders, leading to worse patient outcomes [19,48]. These hypotheses could explain the direct association between sclerostin and the presence of VC, as well as the inverse association between sclerostin levels and CVD events observed in the present study. Still, a study of patients without CKD revealed that sclerostin levels were directly associated with CVD mortality [49]. In addition, sclerostin, which was previously known as a biomarker of bone formation [50], should be paid more attention to by researchers and clinicians for its association with VC and all-cause mortality in patients with CKD.

CKD is incurable and there is a residual risk of adverse events and deterioration. This study, therefore, highlighted the meaning of identifying risk factors of CKD progression and mortality risk. The results also indicate a new insight into the management of CKD and the awareness of serum sclerostin, which might provide evidence to delineate the mechanisms of the stage progression of CKD. Nevertheless, there should be sufficient information about the role of serum sclerostin in VC in patients with CKD, and rigorous validation and demonstration of reproducibility in an independent population are necessary to confirm the impact.

A strength of this study is that it is the first systematic assessment of the association of serum sclerostin with VC and outcome in patients with CKD. Still, it is necessary to consider the limitations of the present meta-analysis while interpreting the results. First, all included studies were observational that inevitably suffered from selection bias, recall bias, no randomization, and often no blinding of the data assessors. Second, limited by the information provided by the included studies, it was not possible to analyze the association of serum sclerostin with VC and outcome according to the CKD stages. Third, the pooled results were limited by the available data and the small number of included studies. The results might be affected by several factors, such as the aging proportions of diabetes cases, and the representativeness might be weakened. Fourth, due to the insufficient information in each study, subgroup analyses could not be performed. Fifth, a potential language bias might exist because the literature search included only articles published in English. Sixth, publication bias cannot be assessed for all analyses because of the small number of studies in some analyses. Seventh, there was significant heterogeneity among studies regarding CKD stage, analyzed vasculature, and VC assessment methods. Finally, this meta-analysis was not registered.

In conclusion, based on the pooled results of 13 studies and 3125 subjects, this meta-analysis suggests that serum sclerostin levels might be associated with VC and all-cause mortality in patients with CKD. Still, considering the heterogeneity observed in analyses, these results must be taken with caution. Carefully designed and performed studies are necessary to confirm these results.

## Ethical approval

This article is a meta-analysis. The data comes from published articles and does not require ethical approval.

## Supplementary Material

Supplemental Material

Supplemental Material

Supplemental Material

## Data Availability

All data generated or analyzed during this study are included in this article. Further inquiries can be directed to the corresponding author.
