# Supplementary material for: Serum sclerostin in vascular calcification in CKD: a meta-analysis
Source: Ren Fail. 2023 Mar 7;45(1):2186151. doi: 10.1080/0886022X.2023.2186151 (PMC10013495; doi:10.1080/0886022X.2023.2186151)
Supplement: Supplemental Material [file IRNF_A_2186151_SM1092.pdf]

Supplementary Table 2. Publication bias and heterogeneity of summarized outcomes

Outcomes	Publication bias	
	Begg (*P* value)	Egger (*P* value)
Summarized relationship between sclerostin and VC	0.46	0.08
Summarized relationship between sclerostin and cardiovascular events	0.62	0.86
Summarized relationship between sclerostin and all-cause mortality	0.32	0.20

Information Classification: General
