# Supplementary material for: Serum sclerostin in vascular calcification in CKD: a meta-analysis
Source: Ren Fail. 2023 Mar 7;45(1):2186151. doi: 10.1080/0886022X.2023.2186151 (PMC10013495; doi:10.1080/0886022X.2023.2186151)
Supplement: Supplemental Material [file IRNF_A_2186151_SM1045.pdf]

Supplementary Table 1. Quality assessment of included studies by Newcastle-Ottawa Scale

NOS case-control Study	Is the case definition adequate?	Representativeness of the cases	Selection of controls	Definition of controls	Comparability of cases and controls on the basis of the design or analysis	Ascertainment of intervention	Same method of ascertainment for cases and controls	Non-response rate	Total quality scores
Claes et al, 2013	☆	☆	-	☆	☆	☆	☆	☆	7
Viaene et al, 2013	☆	☆	-	☆	☆	☆	☆	☆	7
Drechsler et al, 2014	☆	☆	-	☆	☆	☆	☆	☆	7
Gonçalves et al, 2014	☆	☆	-	-	☆	☆	☆	☆	6
Kanbay et al, 2014	☆	-	-	☆	☆	☆	☆	☆	6
Morena et al., 2015	☆	☆	-	-	☆	☆	☆	☆	6
Qureshi et al., 2015	☆	☆	-	☆	☆	☆	☆	☆	7

Information Classification: General

Yang et al., 2015	☆	☆	-	☆	☆	☆	☆	☆	7
Jean et al, 2016	☆	☆	-	☆	☆	☆	☆	☆	7
Kirkpantur et al, 2016	☆	☆	-	☆	☆	☆	☆	☆	7
Wang et al, 2017	☆	☆	-	☆	☆	☆	☆	☆	7
Lips et al, 2017	☆	☆	-	☆	☆	☆	☆	☆	7
Jørgensen et al., 2018	☆	☆	-	-	☆	☆	☆	☆	6
Zhao et al., 2020	☆	☆	-	-	☆	☆	☆	☆	6
Neto et al., 2021	☆	☆	-	-	☆	☆	☆	☆	6

Information Classification: General
