# Supplementary material for: Serum sclerostin in vascular calcification in CKD: a meta-analysis
Source: Ren Fail. 2023 Mar 7;45(1):2186151. doi: 10.1080/0886022X.2023.2186151 (PMC10013495; doi:10.1080/0886022X.2023.2186151)
Supplement: Supplemental Material [file IRNF_A_2186151_SM1008.pdf]

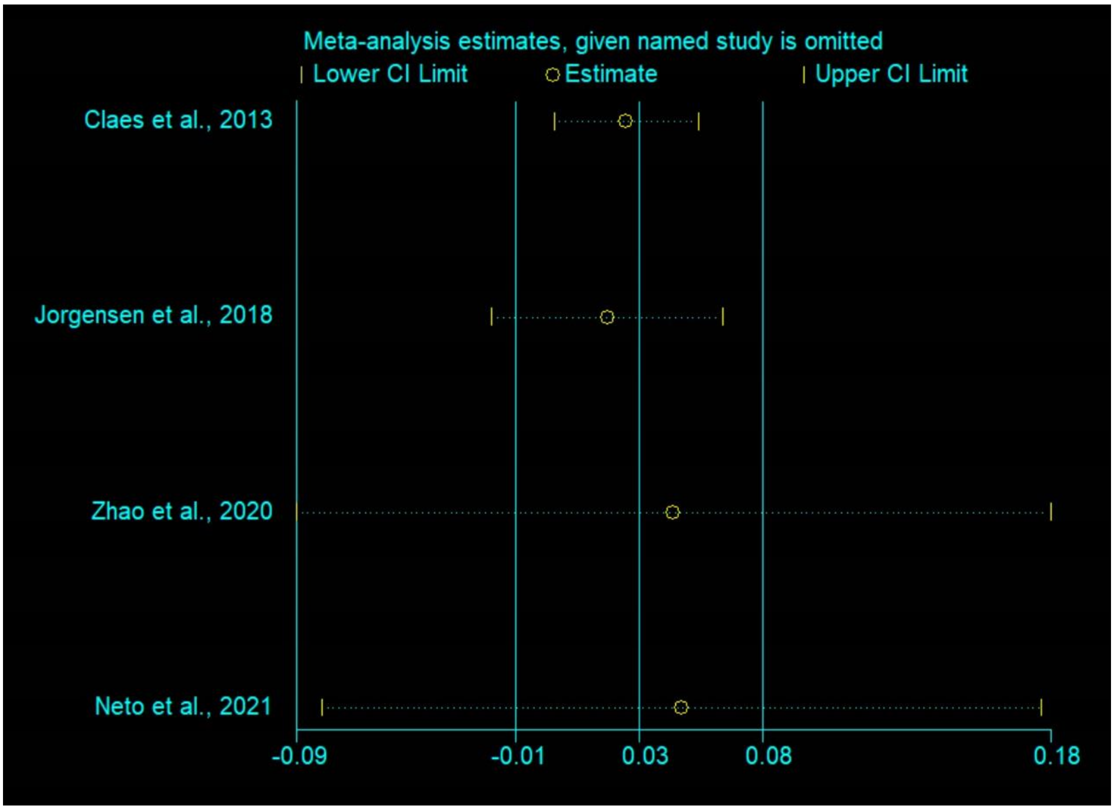

Supplementary Figure 1. Sensitivity analysis on summarized relationship between sclerostin (continuous form) and VC.

Information Classification: General

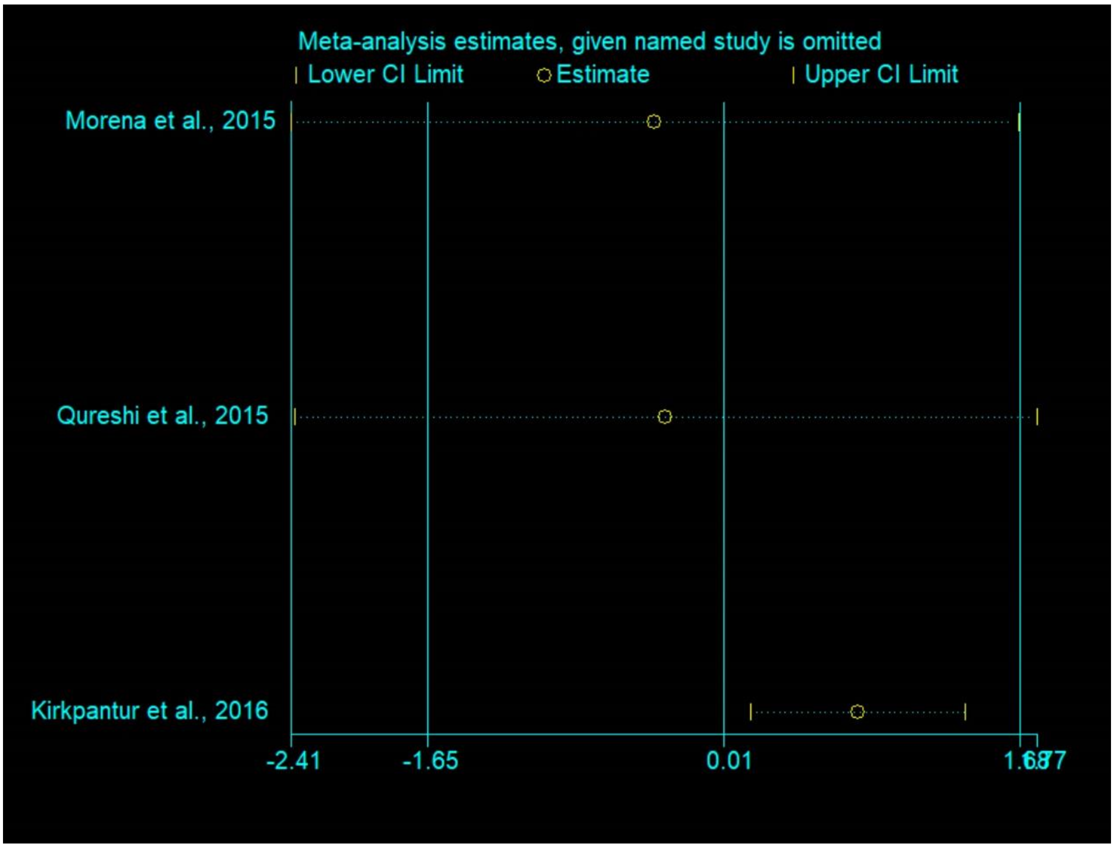

Supplementary Figure 2. Sensitivity analysis on summarized relationship between sclerostin (categorical form) and VC.

Information Classification: General

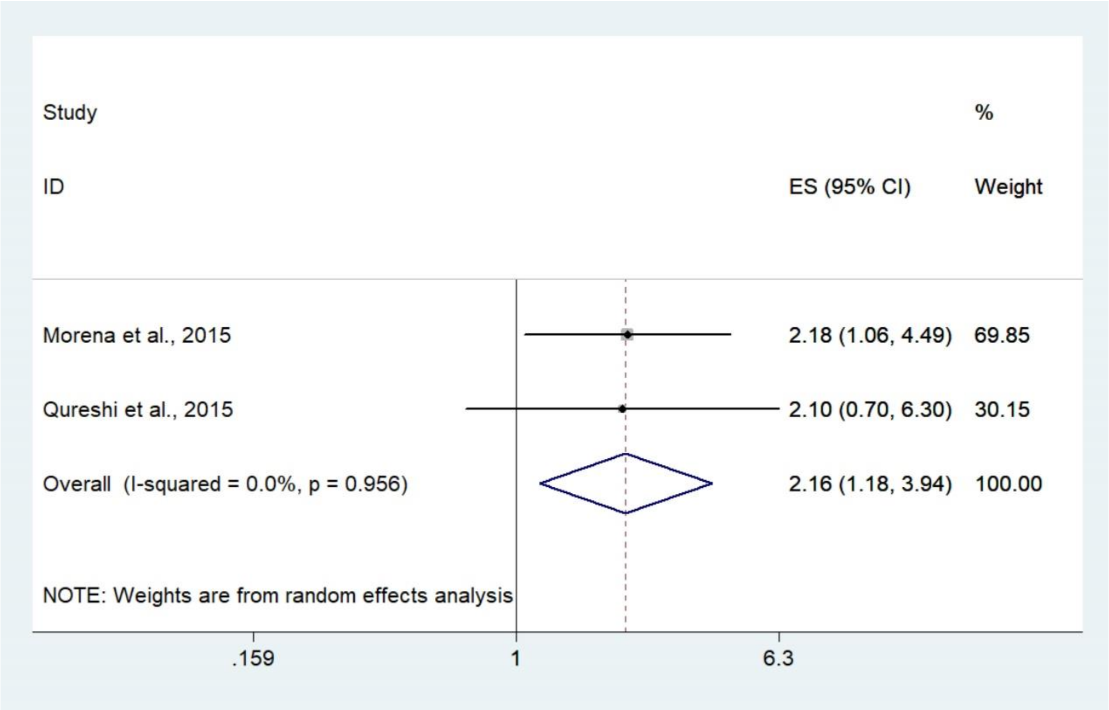

Supplementary Figure 3. Pooled odds ratio for summarized relationship between sclerostin (categorical form) and VC after excluding Kirkpantur et al.

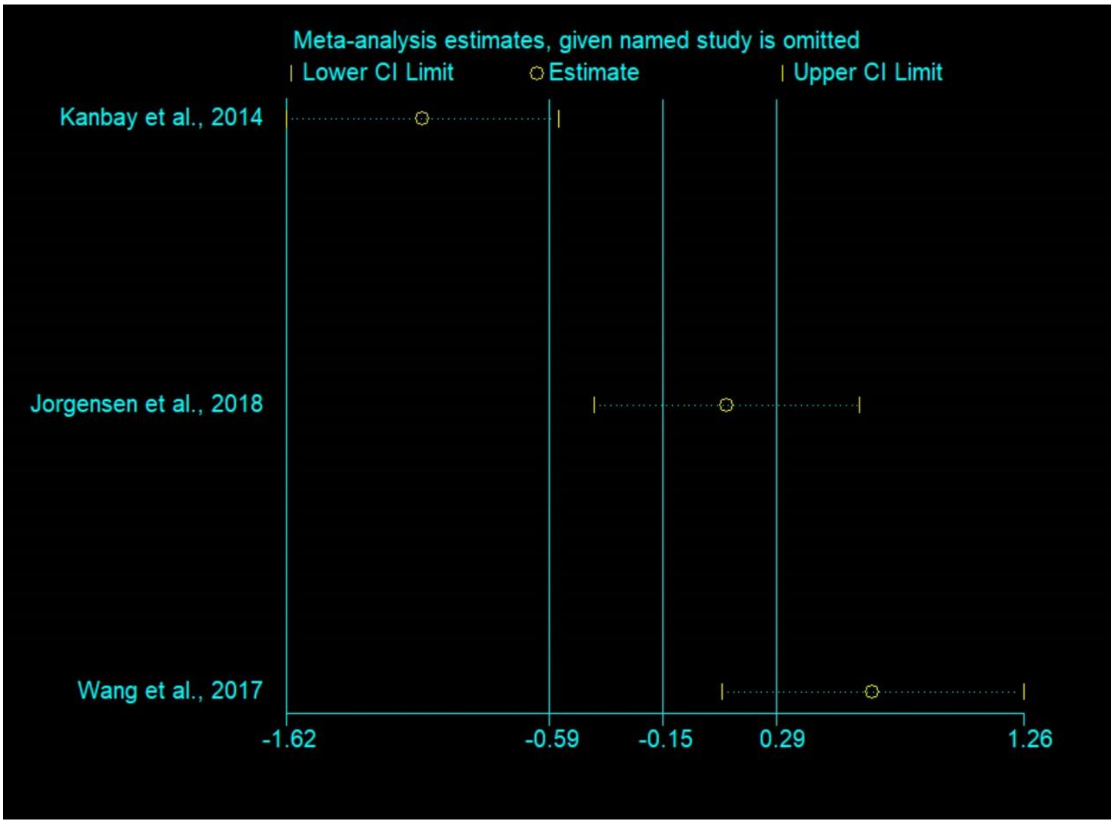

Supplementary Figure 4. Sensitivity analysis on summarized relationship between sclerostin (categorical form) and cardiovascular events.

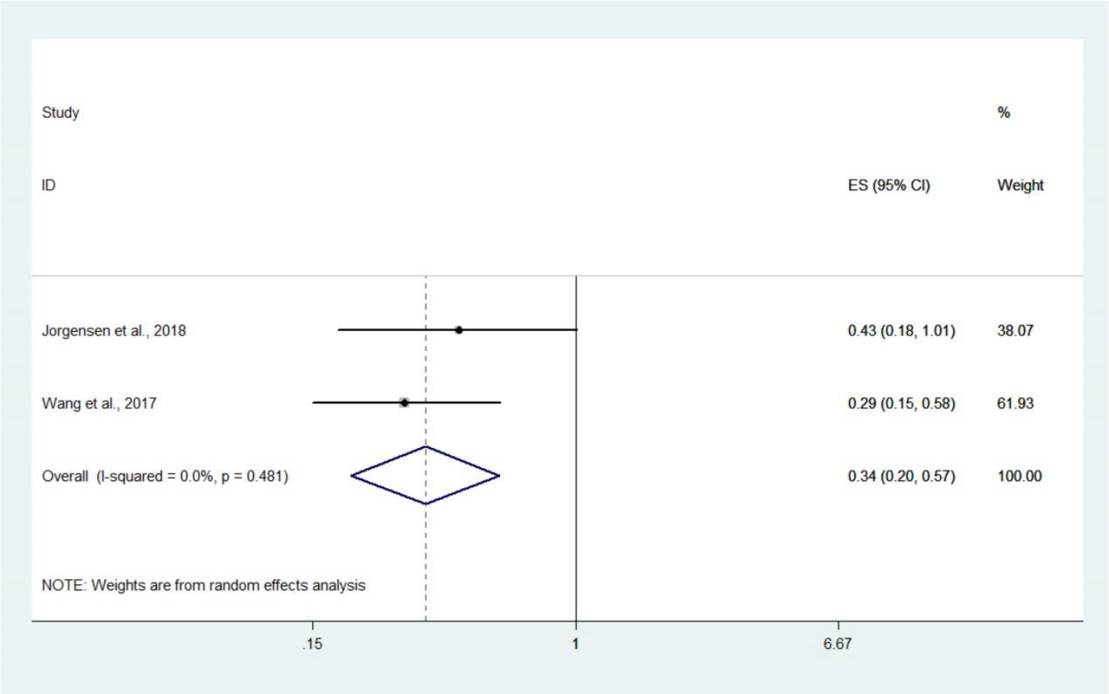

Supplementary Figure 5. Pooled hazard ratio for summarized relationship between sclerostin (categorical form) and cardiovascular events after excluding Kanbay et. al.

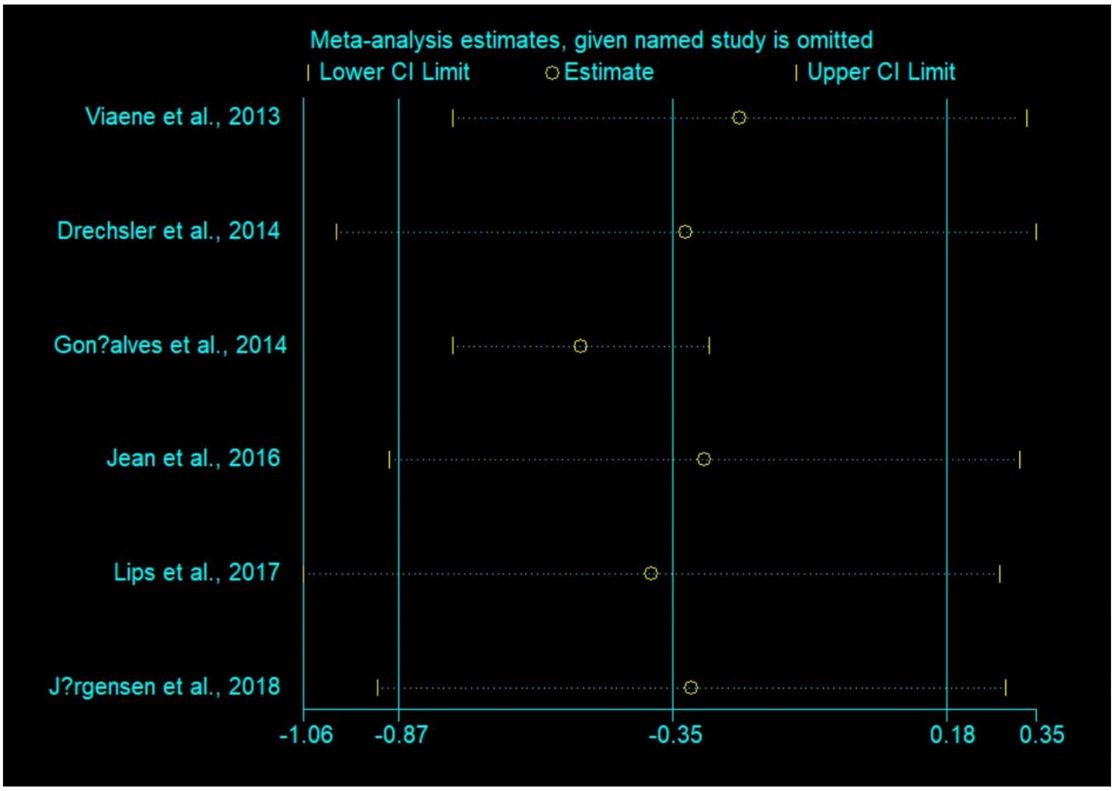

Supplementary Figure 6. Sensitivity analysis on summarized relationship between sclerostin (categorical form) and all-cause mortality.

Information Classification: General

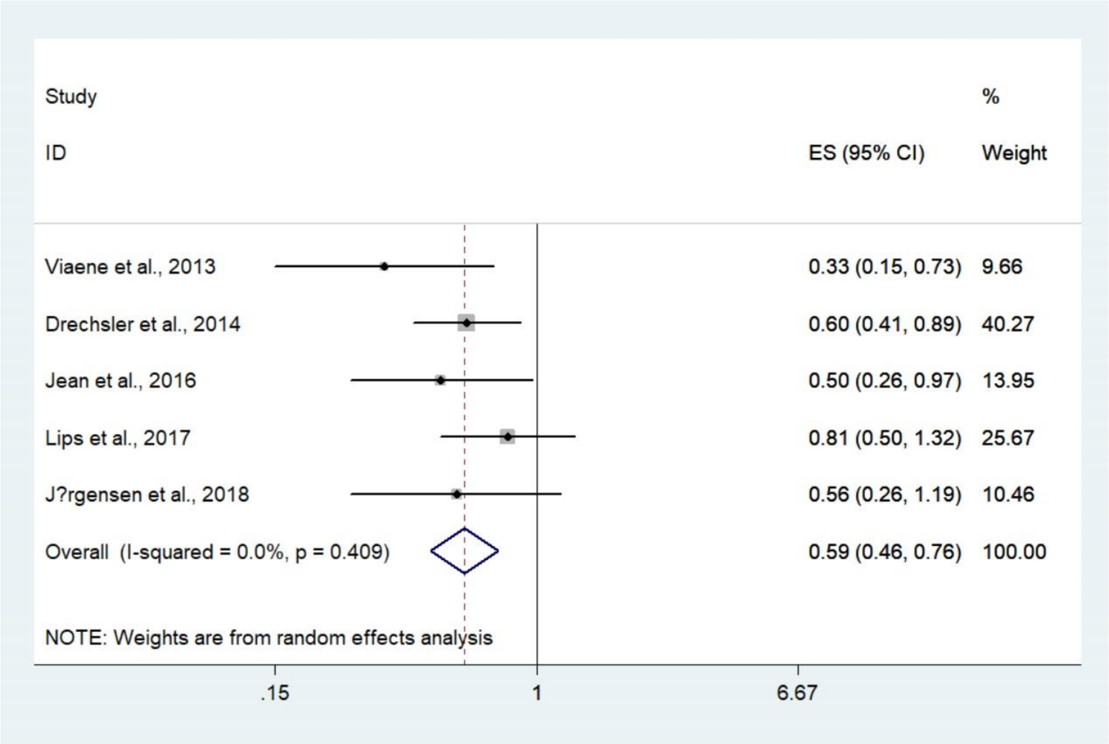

Supplementary Figure 7. Pooled hazard ratio for summarized relationship between sclerostin (categorical form) and all-cause mortality after excluding Gonçalves et al.
